# Evaluation and Validation of a Precision Medicine Platform in Neurodegenerative Disease (PROMINENT)

**DOI:** 10.1002/alz70858_105478

**Published:** 2025-12-25

**Authors:** Lena Sannemann, Hanneke F.M. Rhodius‐Meester, Mats Ekelund, Wiesje M. van der Flier, Kristian Steen Frederiksen, Jean Georges, Maris Hartmanis, Miia Kivipelto, Anne‐Brita Knapskog, Milica G. Kramberger, Peter Lindgren, Juan Domingo Gispert, Jyrki Lötjönen, Sandra Pla, Alina Solomon, Linus Jönsson, Frank Jessen, the PROMINENT consortium

**Affiliations:** ^1^ University of Cologne, Faculty of Medicine and University Hospital Cologne, Department of Psychiatry, Cologne, Germany; ^2^ Department of Geriatric Medicine, The Memory Clinic, Oslo University Hospital, Oslo, Norway; ^3^ Amsterdam Neuroscience, Neurodegeneration, Amsterdam, Netherlands; ^4^ Alzheimer Center Amsterdam, Neurology, Amsterdam UMC Location VUmc, Vrije Universiteit Amsterdam, Amsterdam, Netherlands; ^5^ BioArctic AB, Stockholm, Sweden; ^6^ Danish Dementia Research Centre, Dept. of Neurology, Copenhagen University Hospital ‐ Rigshospitalet, Copenhagen, Denmark; ^7^ Department of Clinical Medicine, Faculty of Health and Medical Sciences, University of Copenhagen, Copenhagen, Denmark; ^8^ Alzheimer Europe, Senningerberg, Luxembourg, Luxembourg; ^9^ FINGERS Brain Health Institute, Stockholm, Sweden; ^10^ Karolinska University Hospital, Theme Inflammation and Aging, Stockholm, Sweden; ^11^ Department of Neurobiology, Care Sciences and Society, Karolinska Institutet, Stockholm, Sweden; ^12^ University Medical Centre Ljubljana and Medical Faculty, University of Ljubljana, Ljubljana, Ljubljana, Slovenia; ^13^ IHE, The Swedish Institute for Health Economics, Lund, Sweden; ^14^ IMIM (Hospital del Mar Medical Research Institute), Barcelona, Spain; ^15^ Barcelonaβeta Brain Research Center (BBRC), Pasqual Maragall Foundation, Barcelona, Spain; ^16^ Universitat Pompeu Fabra, Barcelona, Spain; ^17^ Centro de Investigación Biomédica en Red de Bioingeniería, Biomateriales y Nanomedicina (CIBER‐BBN), Madrid, Spain; ^18^ Combinostics Oy, Tampere, Finland; ^19^ Synapse Research Management Partners SL, Madrid, Spain; ^20^ Institute of Clinical Medicine, University of Eastern Finland, Kuopio, Finland; ^21^ German Center for Neurodegenerative Diseases (DZNE), Bonn, Germany; ^22^ Excellence Cluster on Cellular Stress Responses in Aging‐Associated Diseases (CECAD), University of Cologne, Cologne, Germany

## Abstract

**Background:**

Research advances in diagnostics, therapies and prevention strategies for Alzheimer's disease necessitate a paradigm shift towards timely precision diagnostics and early treatment in routine care. Clinical decision support systems (CDSS) based on artificial intelligence technology have the potential to assist clinicians in the interpretation of complex diagnostic data and facilitate patient management. The PROMINENT study is part of the Innovative Health Initiative (IHI) program and aims to evaluate and validate the use of a CDSS in memory clinics. The tool combines key patient data and uses decision models to provide a probability for the diagnosis and underlying pathology.

**Method:**

The PROMINENT evaluation and validation studies will be carried out at seven European memory clinics. To evaluate the perspectives of different stakeholders on the use of a CDSS, two clinicians, 25 patients and 25 care partners per site will be surveyed, and a subsample will be invited to in‐depth qualitative interviews. A multi‐reader multi‐case design will be applied in the validation study to analyze if the CDSS affects patients’ diagnostic pathways and clinicians’ diagnostic confidence. Two clinicians will retrospectively evaluate the data of 50 patients per site and mimic the real‐world stepwise diagnostic process, with each patient case being assessed once with and without access to the CDSS.

**Result:**

We present the rationale and design of the PROMINENT evaluation and validation studies. The primary endpoint of the evaluation study is the opinion of clinicians on the usefulness of the CDSS and CDSS‐generated reports and the perspective of patients and care partners on the comprehensibility and usefulness of patient‐centered reports. The primary outcome of the validation study is the difference in diagnostic resources required to reach a final diagnosis and the associated diagnostic confidence of clinicians depending on access to the CDSS. In addition, the validation study will assess the performance of the CDSS.

**Conclusion:**

The results of these studies will address the potential of a CDSS to improve diagnostic pathways and clinicians’ diagnostic confidence in memory clinics. Feedback from clinicians, patients, and care partners will inform the tool's integration into routine care, potentially improving decision‐making, quality of care and communication.

